# Supplementary material for: The Omicron Variant Is Associated with a Reduced Risk of the Post COVID-19 Condition and Its Main Phenotypes Compared to the Wild-Type Virus: Results from the EuCARE-POSTCOVID-19 Study
Source: Viruses. 2024 Sep 23;16(9):1500. doi: 10.3390/v16091500 (PMC11437468; doi:10.3390/v16091500)
Supplement: Supplementary file 1 [file viruses-16-01500-s001.zip › viruses-3126874-supplementary.pdf]

viruses MDPI

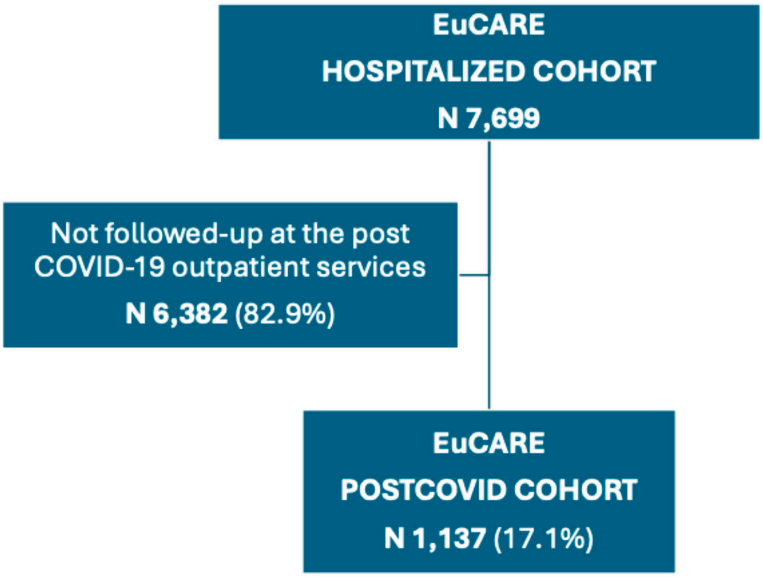

Figure S1. Study flow-chart. Patients admitted to each participating center during the study period were enrolled in the EuCARE hospitalized cohort. Within this cohort, an unselected subgroup of patients underwent at least one follow-up visit over 12–15 months after the acute phase, subsequently entering the EuCARE-POSTCOVID cohort. Participating centers and number of enrolled patients per center in the EuCARE hospitalized cohort: ASST Santi Paolo e Carlo, Milan, Italy (N 3514, 46%); Vilnius University Hospital, Lithuania (N 1536, 20%); Policlinico Tor Vergata, Rome, Italy (N 953, 12%); Regional Hospital Dr. J. Graham Casasús, Mexico (N 631, 8%); Centro Hospitalar de Lisboa Ocidental, Lisbon, Portugal (N 604; 8%); Federal University of Minas Gerais, Belo Horizonte, Brazil (N 461, 6%). Participating centers and number of enrolled patients per center in the EuCARE POSTCOVID cohort: ASST Santi Paolo e Carlo, Milan, Italy (N 643, 48.8%); Vilnius University Hospital, Lithuania (N 62, 4.7%); Policlinico Tor Vergata, Rome, Italy (N 177, 13.4%); Regional Hospital Dr. J. Graham Casasús, Mexico (N 5, 0.4%); Centro Hospitalar de Lisboa Ocidental, Lisbon, Portugal (N 68, 5.2%); Federal University of Minas Gerais, Minas Gerais, Brazil (N 362, 27.5%).

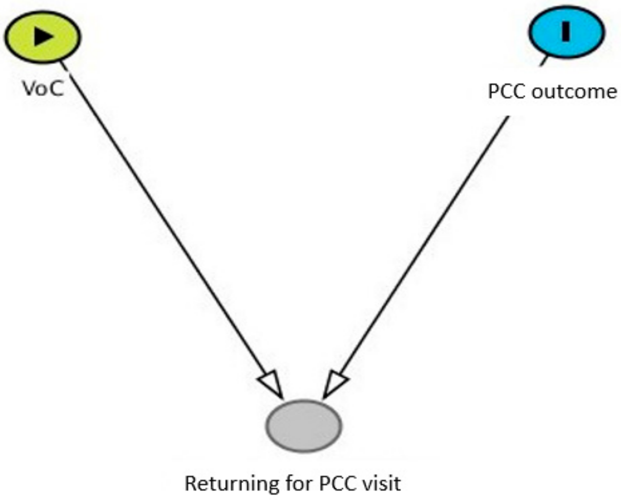

Figure S2. Directed acyclic graph (DAG) of potential collider bias. Directed acyclic graph (DAG) to explore potential collider bias in the association between viral variant (Variants of Concern, VoC; exposure) and post COVID-19 condition (PCC; outcome). By restricting the analysis to those who returned for a post COVID-19 examination it is possible that a spurious association between viral variant and risk of post COVID-19 outcome is introduced.

Table S1. Definition of Post COVID-19 condition (PCC) and main PCC phenotypes.

Definition	
Post COVID-19 condition (PCC)	Symptoms or conditions (ongoing from the acute phase or new onset or fluctuating ones) at ≥1 month after the acute phase.
Brain fog	Memory impairment, troubles in concentrating, attention disorders, forgetfulness, persistent headache, sleep disorders, unrefreshing sleep, sleeping less, sleeping more, cognitive impairment.
Respiratory sequelae	Dyspnea, shortness of breath, persistent dry cough, chest pain.
Fatigue	Fatigue, persistent fatigue, post-exertional malaise.

Definition of Post COVID-19 condition (PCC) according to Centers for Disease Control and Prevention; definition of the main phenotypes of PCC. Symptoms are collected through the WHO Post COVID Case Report Form (CRF).

Table S2. Association between SARS CoV-2 variant and Post COVID-19 condition from fitting a logistic regression model restricted to unvaccinated patients.

	Unadjusted		Adjusted	
**Viral Variant**	**Odds Ratio (95% CI)**	***p*-Value**	**Odds Ratio (95% CI)**	***p*-Value**
	**Unweighted analysis**			
**Wild-type strain**	1	<0.001	1	<0.001
**Alpha**	1.19 (0.83, 1.69)		1.77 (1.13, 2.79)	
**Delta**	0.70 (0.45, 1.11)		0.84 (0.39, 1.81)	
**Gamma**	2.36 (1.10, 5.09)		2.52 (1.08, 5.89)	
**Omicron**	0.24 (0.12, 0.47)		0.12 (0.02, 0.66)	
	**Analysis weighted for viral variant and demographics**			
**Wild-type strain**	1	<0.001	1	<0.001
**Alpha**	1.14 (0.98, 1.33)		1.73 (1.40, 2.13)	
**Delta**	0.69 (0.57, 0.84)		1.06 (0.79, 1.43)	
**Gamma**	2.30 (1.40, 3.79)		2.73 (1.61, 4.63)	
**Omicron**	0.24 (0.20, 0.28)		0.26 (0.15, 0.43)	
	**Analysis weighted for viral variant, demographics and CRP**			
**Wild-type strain**	1	<0.001	1	<0.001
**Alpha**	1.36 (1.13, 1.63)		1.07 (0.81, 1.40)	
**Delta**	0.90 (0.73, 1.11)		0.92 (0.64, 1.31)	
**Gamma**	2.18 (1.04, 4.54)		1.49 (0.68, 3.23)	
**Omicron**	0.17 (0.15, 0.20)		0.23 (0.14, 0.39)	

PCC, Post COVID-19 condition; CI, confidence interval; CRP, C reactive protein. Multivariable analysis is adjusted for age, gender, comorbidities and calendar time of infection (using restricted cubic splines). Unweighted analysis; model 1: weighted analysis for demographic characteristics and variant; model 2: weighted analysis for demographic characteristics, variant and CRP.

Table S3. Association between SARS CoV-2 variant and clusters of symptoms from fitting a logistic regression model restricted to unvaccinated patients.

	OR (95% CI)	*p*	AOR (95% CI)	*p*
**(A) Viral variant and brain fog**	**Unweighted analysis**			
**Wild-type strain**	1	<0.001	1	<0.001
**Alpha**	2.28 (1.65, 3.15)		1.74 (1.12, 2.70)	
**Delta**	1.46 (0.91, 2.32)		0.53 (0.23, 1.22)	
**Gamma**	1.87 (1.06, 3.32)		1.01 (0.51, 2.02)	
**Omicron**	0.60 (0.26, 1.39)		0.04 (0.01, 0.32)	
	**Analysis weighted for viral variant and demographics**			
**Wild-type strain**	1	<0.001	1	<0.001
**Alpha**	2.21 (1.92, 2.54)		1.78 (1.45, 2.19)	

**Delta**	1.37 (1.12, 1.68)		0.83 (0.60, 1.14)	
**Gamma**	1.80 (1.24, 2.63)		1.19 (0.78, 1.80)	
**Omicron**	0.55 (0.45, 0.68)		0.19 (0.10, 0.34)	
	**Analysis weighted for viral variant, demographics and CRP**			
**Wild-type strain**	1	<0.001	1	<0.001
**Alpha**	2.38 (2.04, 2.78)		1.37 (1.08, 1.72)	
**Delta**	1.78 (1.46, 2.16)		0.78 (0.55, 1.11)	
**Gamma**	1.90 (1.12, 3.21)		0.90 (0.51, 1.60)	
**Omicron**	0.34 (0.28, 0.41)		0.13 (0.07, 0.25)	
**(B) Viral variant and respiratory symptoms**	**Unweighted analysis**			
**Wild-type strain**	1	<0.001	1	<0.001
**Alpha**	1.11 (0.81−1.53)		1.17 (0.76−1.79)	
**Delta**	0.52 (0.31−0.86)		0.98 (0.43−2.21)	
**Gamma**	2.27 (1.28−4.00)		1.99 (1.00−3.94)	
**Omicron**	0.28 (0.12−0.67)		1.33 (0.23−7.82)	
	**Analysis weighted for viral variant and demographics**			
**Wild-type strain**	1	<0.001	1	<0.001
**Alpha**	1.09 (0.95−1.25)		1.08 (0.89−1.32)	
**Delta**	0.50 (0.41−0.62)		0.87 (0.63−1.21)	
**Gamma**	2.19 (1.51−3.17)		1.91 (1.27−2.89)	
**Omicron**	0.26 (0.21−0.33)		1.17 (0.65−2.10)	
	**Analysis weighted for viral variant, demographics and CRP**			
**Wild-type strain**	1	<0.001	1	<0.001
**Alpha**	1.37 (1.18−1.60)		1.09 (0.86−1.36)	
**Delta**	0.48 (0.39−0.60)		1.32 (0.92−1.91)	
**Gamma**	3.13 (1.80−5.47)		2.75 (1.50−5.05)	
**Omicron**	0.26 (0.22−0.30)		4.91 (2.77−8.71)	
**(C) Viral variant and fatigue**	**Unweighted analysis**			
**Wild-type strain**	1	<0.001	1	<0.001
**Alpha**	2.49 (1.79−3.47)		3.09 (2.01−4.77)	
**Delta**	1.44 (0.93−2.24)		0.82 (0.37−1.79)	
**Gamma**	0.90 (0.51−1.58)		0.79 (0.40−1.54)	
**Omicron**	0.32 (0.14−0.70)		0.03 (0.00−0.19)	
	**Analysis weighted for viral variant and demographics**			
**Wild-type strain**	1	<0.001	1	<0.001
**Alpha**	2.44 (2.12−2.81)		3.43 (2.80−4.19)	
**Delta**	1.40 (1.16−1.69)		1.58 (1.17−2.15)	
**Gamma**	0.85 (0.59−1.23)		0.99 (0.66−1.49)	
**Omicron**	0.28 (0.23−0.35)		0.18 (0.10−0.33)	
	**Analysis weighted for viral variant, demographics and CRP**			
**Wild-type strain**	1	<0.001	1	<0.001
**Alpha**	2.96 (2.52−3.49)		3.13 (2.48−3.96)	
**Delta**	2.17 (1.78−2.65)		2.36 (1.67−3.33)	
**Gamma**	0.90 (0.54−1.51)		0.88 (0.50−1.55)	
**Omicron**	0.19 (0.16−0.22)		0.27 (0.15−0.48)	

PCC, Post COVID-19 condition; CI, confidence interval; CRP, C reactive protein. Multivariable analysis is adjusted for age, gender, comorbidities and calendar time of infection (using restricted cubic splines). Unweighted analysis; model 1: weighted analysis for demographic characteristics and variant; model 2: weighted analysis for demographic characteristics, variant and CRP.
